# The Plague of 1167

**Published:** 1895-03-16

**Authors:** 


### The Plague of 1167.

The epidemics of the Middle Ages were so numerous that even such indefatigable historians as Hecker and Haeser have been compelled to leave many of them unnoticed. One of the most interesting of these, both from the historic and medical standpoints, is the plague which fell upon the German army in Italy, A.D. 1167, and which has been fully described by several contemporary chroniclers. The following outline is based mainly on the account given by Henry, Archdeacon of Salzburg, who says that he has carefully compiled it from the narratives of eye-witnesses.

The long controversy between the Empire and the Papacy had culminated in an open schism and the establishment of an Anti-pope, to support whom Frederick Barbarossa invaded Italy with a great army directed at once against the independence of the Church and of the Lombard cities. The terrible destruction of this army in the moment of its triumph sent a thrill of mingled horror and exultation throughout Europe.

"Then happened a thing unheard of since the creation of the world, which till the end thereof will make the ears of whoso heareth it to tingle. Peruse the chronicles of the nations; where will you find a tale such as that which tells how the power, wealth, and pride of all Germany died, not as men die, neither fell like one of the princes, but were destroyed in heaps, like the rain-worms or the locusts, after a tempest?" The great church of St. Peter at Rome, had been taken by storm. "They broke down the gates thereof with axes and swords, and cut off chips from the doors, boasting that they would take them to their wives as tokens of their own powers, and of the ignominy of the blessed Peter; so the vengeance of God came upon them in the pestilence." Three days later, August 1st, Frederick and Beatrice were crowned with great pomp by the Anti-pope Paschalis, in the still blood-stained church. The next morning was marked by a sudden tempest of thunder and rain, after which the sun shone out brightly, till a thin mist rising from the ground covered the tents of the schismatic army, and then the plague began. The number of the victims, says Henry, great as it was, was less terrible than the suddenness of their deaths. There were a hundred chosen men in armour (loricati) who formed the Emperor's body guard, and camped apart from the rest. "The Emperor had mounted his horse, and, wondering at the delay of his guards, sent a messenger for them. He found their weapons piled, and their horses tethered, but the hundred men in armour and their attendants were all of them dead corpses!" The plague seemed specially to single out the great men of the army, particularly the schismatic clergy. Rainald, Archbishop of Cologne, "head and leader of the schism," the Bishops of Prague, Regensburg, Spires, Verdun, and Liège, all died in a few days, and the death of so many princes and nobles brought into prominence, if it did not originate, a curious custom of some interest for the history of anatomy. Many persons, "ashamed to leave their dead friends in a hostile country," boiled their bodies, and extracted the bones in order to take them home to their fatherland. Thus they boiled Bishop Daniel and took his bones to Prague, but his clergy refused to pray for his soul, till it appeared to one of them and told him he had repented of his schism before death. Some, according to Henry, tried to preserve the whole body by cooking and salting it. "A certain man was cooking his brother in a cauldron, when a friend sent to borrow it for a similar purpose, but he replied that he could not have it, for after cooking his brother he should want to be cooked in it himself, which was also done."

The disease seems to have been a very malignant form of ague. There were no eruptions or inflammations, though in some of the fatal cases "dark markings appeared between the shoulders." Those who recovered were left with a peculiar feebleness and pallor, and a similar weakness came upon the beasts of burden, which were no longer able to carry sufficient food to the camp. Famine seemed likely to be added to the pestilence, when, on August 6th, Frederick gave orders for the retreat; he had lost seven thousand men in four days. In the passage of the Apennines there died two thousand more, "slain by the sword of God." Multitudes were killed by the hostile guerilla bands, for they had thrown away their arms, either through feebleness or that they might better carry their sick comrades, while the few remaining horses and mules were laden, not, as had been fondly hoped, with the spoil of Rome, but with the boiled skeletons of bishops and princes. The Emperor himself was taken ill, but recovered on being bled by the surgeons, by whose advice all the survivors had their heads shaved, for they thought the contagion might be carried in the hair. Finally, on September 12th, a band of pallid and tottering scarecrows, the scanty remnant of the proudest army that had ever marched against Rome, sheltered itself behind the walls of the faithful city of Pavia, where they were refreshed "with baths and medical diet."

Such a disaster could not fail to be attributed to the special action of the Deity. The Emperor himself confessed that it was probably a punishment for his shortcomings, and the only doubt in the minds of ecclesiastical writers was whether he could best be compared with Pharaoh or with Sennacherib. As soon as Thomas à'Becket heard the story, he wrote to congratulate the Pope on the destruction of the Assyrians. Sennacherib, indeed, had escaped, reserved, doubtless, for greater judgments. "May the Lord make him a spectacle to the nations, that men may point at him and say: 'Lo! this is the man who took not God for his help, &c.'" "The Lord hath crushed Frederick, the hammer in the hands of the ungodly," exclaims the archbishop elsewhere. Frederick, however, by no means considered himself crushed, and hurling his gauntlet from the walls of Pavia, he bade defiance both to the Pope and the Lombard League. Or, in the language of Archdeacon Henry, "What is more terrible and pitiful than all these disasters, of which but a small part has been told you, is the fact that Pharaoh's heart was hardened, so that neither the miserable deaths of his friends, relatives, and great princes, nor the destruction of so glorious an army could persuade him to recede from his schism, and to love and reverence Holy Mother Church as his own."

